# A Reaction-Diffusion-Based Coding Rate Control Mechanism for Camera Sensor Networks

**DOI:** 10.3390/s100807651

**Published:** 2010-08-13

**Authors:** Hiroshi Yamamoto, Katsuya Hyodo, Naoki Wakamiya, Masayuki Murata

**Affiliations:** Graduate School of Information Science and Technology, Osaka University, 1-5 Yamadaoka, Suita, Osaka 565-0871, Japan; E-mails: k-hyodo@ist.osaka-u.ac.jp (K.H.); wakamiya@ist.osaka-u.ac.jp (N.W.); murata@ist.osaka-u.ac.jp (M.M.)

**Keywords:** camera sensor network, coding rate control, reaction-diffusion model

## Abstract

A wireless camera sensor network is useful for surveillance and monitoring for its visibility and easy deployment. However, it suffers from the limited capacity of wireless communication and a network is easily overflown with a considerable amount of video traffic. In this paper, we propose an autonomous video coding rate control mechanism where each camera sensor node can autonomously determine its coding rate in accordance with the location and velocity of target objects. For this purpose, we adopted a biological model, *i.e.*, reaction-diffusion model, inspired by the similarity of biological spatial patterns and the spatial distribution of video coding rate. Through simulation and practical experiments, we verify the effectiveness of our proposal.

## 1. Introduction

By distributing a large number of sensor nodes with wireless communication capability and organizing a wireless sensor network (WSN), we can obtain detailed information about surroundings, remote region, and objects [1]. Sensor nodes are equipped with a variety of sensors depending on applications. In particular, a camera sensor network, which is composed of sensor nodes equipped with a camera, are useful in applications such as remote surveillance and monitoring [2] and thus it is one of key technologies for the safe and secure living environment. For example, by placing camera sensor nodes along streets, we can monitor the traffic condition, children on the way to and from a school, or track suspicious people.

Generally, control mechanisms for sensor networks must be scalable, adaptive, and robust, because of a large number of sensor nodes, random or unplanned deployment, and dynamic topology changes due to addition, movement, and removal of sensor nodes. In addition, due to difficulty in managing a large number of nodes in a centralized manner for the limited bandwidth insufficient to periodically collect the up-to-date information from nodes, mechanisms must be fully distributed and self-organizing. Camera sensor networks place additional challenging issues as summarized in [3]. In particular, compared to typical scalar and even analog sensor data such as humidity, temperature, and acceleration, the volume of video data generated by cameras are considerably large, ranging from a few hundred Kbps to several hundred Mbps. The capacity of wireless sensor networks is limited to accommodate such huge video traffic. For example, the communication rate of ZigBee with IEEE 802.15.4 [4], a standard protocol for WSN, is only 250 Kbps. Even when we adopt IEEE 802.11b with the capacity of 11 Mbps, theoretical analysis indicates that the throughput available to each node is in the order of $O\left(\frac{1}{\sqrt{n}}\right)$, where *n* is the number of nodes in the multi-hop wireless network [5]. Therefore, only a few nodes can join the network, even when each node transmits video data coded at only a few Mbps using IEEE 802.11b. Many studies, such as bandwidth allocation, retransmission control [6], FEC (Forward Error Correction) [7], and other error concealment techniques [8] have been made for QoS control for video transmission in wireless networks. They are useful and effective to maintain the quality of video data when a wireless network is lightly loaded and moderately congested, but they do not help much when the volume of video traffic is, for example, twice as much as the capacity of the network.

Therefore in large scale camera sensor networks, in addition to these QoS control mechanisms, application-level control to reduce the amount of video traffic without impairing the application is required. When we consider surveillance and monitoring applications, not all video data from cameras are equally important. Users are interested in video images that capture targets or certain phenomena. Therefore, the quality of video data from nodes detecting targets should be as high as possible without overwhelming the wireless network capacity, whereas those nodes far from the targets can suppress the video coding rate to avoid wasting the wireless network capacity in the transmission of irrelevant and redundant video data. For this purpose, we need a mechanism for camera sensor nodes to adjust the video coding rate in accordance with the location and velocity of targets. To the best of our knowledge, such application-level control has not been addressed well and we can only find a few [9][10]. In [9], a content-aware control system assigning higher rate to cameras capturing important images is proposed. However, they mainly consider bandwidth allocation among cameras on a node, not among camera nodes. In [10], the authors propose dynamic allocation of wireless communication bandwidth to camera nodes based on the rate-distortion model and the activity of captured video. However, the bandwidth allocation is performed in a centralized manner, where a central location has the complete information about the status of wireless network and characteristics of video data at all cameras.

Only if we know the complete and up-to-date information about the location and status of camera sensor nodes and the location and velocity of targets, we can optimally assign the wireless network capacity to those nodes and set the video coding rate to avoid congestion of the network while keeping the application-level QoS. However, maintenance of such global and up-to-date information involves much communication overhead and the battery power of nodes. Therefore, an autonomous, distributed, and self-organizing mechanism is required for each node to appropriately determine the video coding rate based on local information obtained by exchanging messages, e.g., general HELLO messages, with neighboring nodes, while avoiding local network congestion.

To accomplish the above mentioned goal, we adopt a reaction-diffusion model in this paper. A reaction-diffusion model was first proposed by Dr. Alan Turing as a mathematical model for pattern generation on the surface of body of fishes and mammals [11]. In a reaction-diffusion model, through local interactions among molecules of neighboring cells, a variety of spatial patterns, *i.e.,* heterogeneous distribution of morphogen concentrations emerge in a self-organizing manner. Autonomously generated patterns can be used for routing, clustering, scheduling, and topology control on sensor networks [12–15]. In smart sensor networks for a forest fire application, a stripe pattern is organized from a robot load point to a fire control point through local and mutual interactions among distributed sensor nodes and mobile robots walk along the stripe to fight a fire [12]. RDMAC [13] is a reaction-diffusion based MAC protocol, where they noticed the similarity among a scheduling pattern of spatial TDMA and a spot pattern of leopards. A node inhibits packet emission of neighboring nodes in its range of radio signals while encouraging nodes out of the range to send packets for better spatial use of a wireless channel. For camera sensor networks, a cooperative control model for a surveillance system which consists of plural Pan-Tilt-Zoom cameras and having no central control unit is proposed [15]. Each camera adjusts their observation area to decrease blind spots in the whole surveillance area by control algorithms based on a reaction-diffusion model. Our early work on a reaction-diffusion based control mechanisms for a sensor network [14] verified the practicality of reaction-diffusion based pattern formation on a wireless sensor network under the influence of collisions through experiments and two acceleration schemes for faster pattern generation were proposed.

In this paper, we propose an autonomous mechanism based on a reaction-diffusion model for coding rate control in camera sensor networks for remote surveillance and tracking applications. Specifically, we generate spot patterns of concentrations of virtual morphogens so that the morphogen concentrations have a peak at a camera detecting a target in its observation area. In addition, to prepare cameras in the direction of a moving target for future detection, their video coding rates, in other words, their morphogen concentrations are made slightly higher than others. As a result, we see a pattern of concentric circles for a stationary target and an elliptic pattern for a moving target (see Figure 3). Furthermore, we also propose a mechanism to keep the total of coding rate low for closely located targets considering the limited capacity of a wireless network. First, through simulation experiments, we verified the effectiveness of our proposed mechanism. Then we implemented our proposed mechanism on a wireless sensor network composed of PCs with cameras and conducted practical experiments. Through practical experiments, we verified that the video coding rate was appropriately adjusted in accordance with the location and velocity of a moving target. We also evaluated the effectiveness of the mechanism in avoidance of congestion and the perceived quality of received video data.

The rest of the paper is organized as follows. In Section 2, we introduce a pattern generation mechanism of a reaction-diffusion model that our paper is based on. Next in Section 3, we describe our reaction-diffusion based control mechanism for camera sensor networks. In Section 4, we then show and discuss results of simulation experiments. In Section 5, we describe our implementation of the mechanism and show and discuss results of practical experiments. Finally, we conclude the paper in Section 6.

## 2. Pattern Formation by Reaction-Diffusion Model

**Figure 1.**
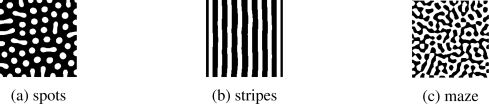
Example of patterns generated by reaction-diffusion model.

In a reaction-diffusion model, by mutual interaction among neighbor cells through chemical reaction and diffusion of two morphogens, spatially heterogeneous distribution of concentration of morphogens appears. Depending on the form of reaction-diffusion equations and their parameters, a variety of patterns can be generated as illustrated in Figure 1.

Generally, a reaction-diffusion model is formulated by a pair of partial differential equations as follow.

(1) $$\{\begin{array}{l} \frac{\partial u}{\partial t}=F\left(u,v\right)+D_{u}\nabla^{2}u\\ \frac{\partial u}{\partial t}=G\left(u,v\right)+D_{u}\nabla^{2}v, \end{array}$$

where *u* and *v* are the concentrations of activator and inhibitor, respectively. *D_u_* and *D_v_* are the diffusion rate of activator and inhibitor respectively. *F* and *G* are functions for reactions. ▿^2^ is the Laplacian operator. The first term of the right-hand side is called a reaction term and the second term is called a diffusion term. In the reaction-diffusion model, the following two conditions must be satisfied to generate patterns. First, the activator activates itself and the inhibitor, whereas the inhibitor restrains itself and the activator. Second, the inhibitor diffuses faster than the activator (*D_v_* > *D_u_*).

**Figure 2.**
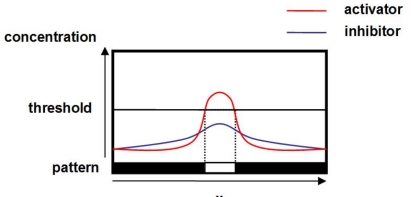
Concentration distribution of activator and inhibitor.

A mechanism of pattern generation can be explained as follows. In Figure 2, those hypothetical chemicals are arranged in a line on the horizontal axis. The vertical axis corresponds to the concentrations of activator and inhibitor. Now consider that the concentration of activator has the peak at the point *x* illustrated in Figure 2, by a slight perturbation. The concentrations of activator and inhibitor are increased around the point *x* by being activated by the activator. The generated inhibitor diffuses faster than the activator and restrains generation of activator at further regions. On the other hand, at the point *x*, because of the different rate of diffusion, the activator stays and the concentration of activator is kept higher than that of inhibitor. Consequently, the diversity in the concentration of activator emerges and a pattern appears. For example, when we color points where the concentration of activator exceeds a certain threshold with white and others with black, we can see a black-while-black pattern shown at the bottom of Figure 2.

In this paper, since we want to generate spot patterns where spots correspond to targets, we introduce the *stimulus* which is an increment of activator and apply the following reaction-diffusion equation.

(2) $$\{\begin{array}{l} \frac{\partial u}{\partial t}=F\left(u,v\right)+D_{u}\nabla^{2}u-\mathrm{du}+E\left(t\right)\\ \frac{\partial v}{\partial t}=G\left(u,v\right)+D_{v}\nabla^{2}v-\mathrm{gv}, \end{array}$$

and

(3) $$\{\begin{array}{l} F\left(u,v\right)=\text{max}\{0,\text{min}\left\{\mathrm{au}-\mathrm{bv}-c,M\right\}\}\\ G\left(u,v\right)=\text{max}\left\{0,\text{min}\left\{\mathrm{eu}-\mathrm{hv}-f,N\right\}\right\}, \end{array}$$

where *d* and *g* are parameters for decomposition or decrease of morphogens per unit time. *E*(*t*) is the amount of stimulus. Intentionally placed activator, *i.e.*, *E*(*t*), increases the activator concentration at the point and a spot centered at the point would emerge. *a* and *e* correspond to the rate of activation and *b* and *h* are for inhibition. *c* and *f* are parameters for decrease of morphogens per unit time. *M* and *N* are constants of limit.

Theoretically speaking, these parameters must satisfy the following conditions to generate a pattern centered at a point where stimulus *E*(*t*) > 0 exists [16].

(4) $$\frac{a-d}{b}\frac{f}{e}-\frac{c}{b}\leq 0\;\text{and}\;\frac{a-d}{b}\frac{M}{d}-\frac{c}{b}<\text{min}\left\{\frac{e}{h+g}\left(\frac{M}{d}-\frac{f}{e}\right),\frac{N}{g}\right\}$$

or

(5) $$a-d>h+g\;\text{and}\;\frac{a-d}{b}\frac{M}{d}-\frac{c}{b}<\text{min}\left\{\frac{e}{h+g}\left(\frac{M}{d}-\frac{f}{e}\right),\frac{N}{g}\right\}$$

As can be seen, the parameter setting does not depend on system conditions such as the size of region.

## 3. Reaction-Diffusion Based Coding Rate Control Mechanism

**Figure 3.**
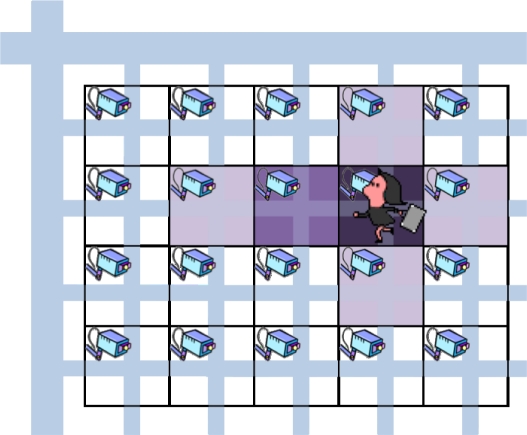
Camera sensor network.

Figure 3 illustrates a surveillance or monitoring system that we consider in this paper. Each square corresponds to the observation area of a camera sensor node. The darker the square is, the higher the video coding rate is. We assume that nodes are arranged in a grid topology, considering town or room monitoring as an application of the mechanism. For example in a town, we can consider such a scenario where camera sensor nodes are placed at intersections. A node can communicate with four neighbors in up, right, down, and left directions. Nodes at a corner of the monitoring region have two neighbors and nodes at an edge have three neighbors. The assumption on node layout can be relaxed by further discretization ▿^2^*X*(*X* = *u, v*) as follow.

(6) $$\nabla^{2}X=\sum_{j\neq i}k_{i,j}\left(X_{j}-X_{i}\right)$$

(7) $$k_{i,j}=\{\begin{array}{ll} d_{i,j}^{-2}, & d_{i,j}\leq R,\\ 0, & \text{otherwise} \end{array}$$

where, *X_i_* and *X_j_* are the concentration of morphogens in node *i* and *j*, and *d_i,j_* is the distance between the two nodes. *R* is the maximum transmission range.

Each node has a camera and a wireless communication device. A camera or a node has the capability of object recognition and motion detection with which the existence, speed, and direction of a target in its observation area are recognized.

### 3.1. Basic Behavior

Basically, at regular control intervals of *T* seconds, each node calculates the reaction-diffusion equation by using the information it received in the preceding control interval, adjusts its video coding rate in accordance with morphogen concentrations, and then broadcasts a message containing information about its morphogen concentrations, stimulus *E*, attenuation coefficient *A*, and NIP (Notification of Inhibitor Peak) notification to its neighbors. Message emission is done once per control interval and the message can be combined with a general HELLO message. We call the duration between the *t*-th control timing and the *t* + 1-th control timing as the *t*-th control interval. We should note here that nodes behave in an asynchronous manner, although the control interval is identical among nodes. It means that timing of reaction-diffusion calculation and message emission is different among nodes.

The reaction-diffusion equation is identical among nodes. Since nodes are arranged in a grid layout and messages are exchanged at regular intervals, we spatially and temporally discretize Equations (2) and (3) as follows.

(8) $$\{\begin{array}{l} u_{t}=u_{t-1}+\Delta t\{F\left(u_{t-1},v_{t-1}\right)-du_{t-1}+E\left(t-1\right)\\ \;+D_{u}\frac{\left(u_{t-1}^{u}+u_{t-1}^{d}+u_{t-1}^{l}+u_{t-1}^{r}-4u_{t-1}\right)}{\Delta h^{2}}\}\\ v_{t}=v_{t-1}+\Delta t\{G\left(u_{t-1},v_{t-1}\right)-gv_{t-1}\\ \;+D_{v}\frac{\left(v_{t-1}^{u}+v_{t-1}^{d}+v_{t-1}^{l}+v_{t-1}^{r}-4v_{t-1}\right)}{\Delta h^{2}}\}, \end{array}$$

and

(9) $$\{\begin{array}{l} F\left(u_{t-1},v_{t-1}\right)=\text{max}\left\{0,\text{min}\left\{au_{t-1}-bv_{t-1}-c,M\right\}\right\}\\ G\left(u_{t-1},v_{t-1}\right)=\text{max}\left\{0,\text{min}\left\{eu_{t-1}-hv_{t-1}-f,N\right\}\right\}. \end{array}$$

At the *t*-th control timing, a node calculates the above reaction-diffusion equation to derive its morphogen concentrations *u_t_* and *v_t_*. A set of $u_{t-1}^{u}$, $u_{t-1}^{r}$, $u_{t-1}^{d}$, and $u_{t-1}^{l}$ and a set of $v_{t-1}^{u}$, $v_{t-1}^{r}$, $v_{t-1}^{d}$, and $v_{t-1}^{l}$ correspond to concentrations of activator and inhibitor of neighboring nodes in up, right, down, and left directions. These values are obtained from messages that a node received in the *t* − 1-th control interval. If a node did not receive a message from a neighboring node in the *t* − 1-th control interval, the latest value obtained in the preceding intervals is used instead. Δ*h* and Δ*t* correspond to the distance between nodes and the discrete step interval of time, respectively. There is the theoretical range of Δ*t* for the equation reaches convergence and a stable pattern is formed.

(10) $$\begin{array}{r} 0<\Delta t<\text{min}\{\frac{2}{d+4D_{u}\left(\Delta x^{-2}+\Delta y^{-2}\right)},\\ \frac{2}{g+4D_{v}\left(\Delta x^{-2}+\Delta y^{-2}\right)}\}. \end{array}$$

If the degree of temporal discretization is not within this range, a pattern does not converge. *E*(*t* − 1) in Equation (8) is the amount of stimulus which is determined at the *t*-th control timing, based on messages it received in the *t* − 1-th control interval and the condition of a target if exists. The stimulus controls the distribution of morphogen concentrations, that is, a pattern. Usually, the amount of stimulus is zero. A node which detects a target in the *t* − 1-th control interval appropriately sets *E* and *A* in accordance with the speed and direction of the target, so that a spot pattern centered at the node is generated. The stimulus diffuses to nodes in the direction of target movement so that they prepare for the future appearance of the target in their observation area as shown in Figure 3. The attenuation coefficient *A* is used for this purpose. Details of stimulus determination and diffusion will be explained in Section 3.2. NIP notification is used to regulate the amount of stimulus when two or more targets are closely located. Details of NIP will be given in Section 3.3.

**Figure 4.**
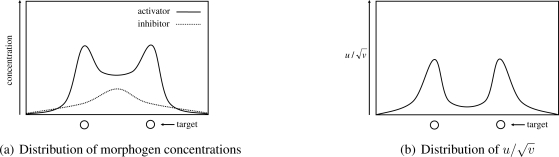
Distribution of *u*, *v*, and $u/\sqrt{v}$ with closely located targets.

Once morphogen concentrations are derived, a node translates the concentrations to the video coding rate that it uses during the *t*-th control interval. As explained in Section 2, a spatial pattern generated by a reaction-diffusion model comes from the spatial heterogeneity in concentration of activator. However, an approach to directly map the concentration of activator to the video coding rate fails when two or more targets are close together. In Figure 4(a), we illustrate the distribution of concentrations of morphogens. As can be seen, the region between two peaks of the concentration of activator has the slightly high concentration of activator, because the region is activated by diffused activator while it is also inhibited by diffused inhibitor. If we set the video coding rate in proportional to the concentration of activator for example, regions in between closely located targets generate unnecessarily high-quality video data. Therefore, in our mechanism, by focusing on a phenomenon that the concentration of inhibitor is also high in the region as shown in Figure 4(a), a node determines the video coding rate based on $u/\sqrt{v}$, whose distribution is illustrated in Figure 4(b). If *v* < 1, then we set $u/\sqrt{v}=0$, in order to avoid the divergence of $u/\sqrt{v}$. In the paper, we determine the video coding rate based on the value $u/\sqrt{v}$.

### 3.2. Stimuli Arrangement

In the reaction-diffusion equation, stimuli decide the position, shape, and size of spot patterns. When we set the stimulus high at a certain node, the concentration of activator becomes high at the node and a spot centered at the node emerges. To keep monitoring a moving target, cameras in the region to which the target is expected to move should use the sufficiently high coding rate for the future appearance (Figure 3). Therefore, in our mechanism, a node detecting a moving target diffuses the stimulus to nodes in the moving direction while the stimulus decreases as the distance to the target increases.

If a node has a target in its observation area at the control timing, it sets the amount of stimulus *E*, the attenuation coefficient *A*, and the direction of diffusion. The node calculates the morphogen concentrations, set the video coding rate, and broadcasts a message containing this information. A node receiving a message first sees whether it is in the direction of the target movement. If not, it ignores the information. If the node should be prepared for the target, it first calculates the amount of stimulus *E*′ from the informed *E* and *A* as *E*′ = *A* × *E*. This stimulus is used as *E*(*t* − 1) at the *t*-th control timing in calculating the morphogen concentrations. After the calculation, the information about the stimulus, including *E*′, *A*, and the direction is further diffused to neighbor nodes by being embedded in a broadcast message. When a node receives multiple messages containing stimuli from neighbor nodes, it uses the sum of *E*′ as *E*(*t* − 1).

**Figure 5.**
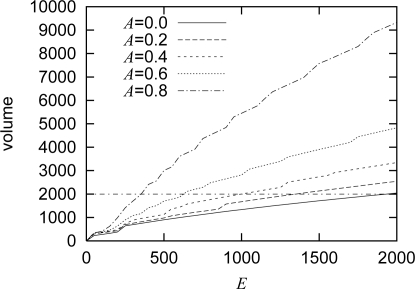
Relationship among *A*, *E*, volume of pattern.

The amount of stimulus *E* and the attenuation coefficient *A* are determined in accordance with the movement of target and the capacity of wireless channel. In Figure 5, the relationship among *E*, *A*, and the volume of a generated pattern is shown for the case with a single target. The volume of a pattern is defined as,

(11) $$v\left(E,A\right)=\sum_{\left(i,j\right)}u\left(i,j\right)/\sqrt{v\left(i,j\right)}.$$

*u*(*i, j*) and *v*(*i, j*) are the concentrations of activator and inhibitor at node (*i, j*) on a converged stable pattern, respectively. Since a node chooses the video coding rate depending on $u\left(t\right)/\sqrt{v\left(t\right)}$, the volume corresponds to the total amount of traffic generated by nodes in a spot. As shown in the figure, the volume *v*(*E, A*) is almost in proportional to the stimulus *E*. As the attenuation coefficient *A* increases, the volume *v*(*E, A*) increases for the same stimulus *E*. Since the attenuation coefficient *A* determines the range of stimulus diffusion, a node detecting a target first sets the attenuation coefficient *A* in accordance with the speed *V* of target. Then, the node determines the stimulus *E* to keep the total traffic at a certain volume from *v*(*E, A*). For example, when the capacity of local wireless network is 2,000 in volume and *A* is 0.4, *E* is set at 1,010. The mapping from the speed to the attenuation coefficient depends on the system conditions and application requirements. When the distance between adjacent nodes is large, the attenuation coefficient should be large not to spread a spot pattern too broadly and thus not to exceed the capacity of local wireless network. Regarding mapping from the network capacity to the volume, we suggest to use 2,000 for any capacity as far as actual video coding rate is adjusted accordingly. In our experiments we use MPEG-2 as a coding algorithm and the coding rate ranges from 0.75 Mbps to 2 Mbps for an IEEE 802.11g network. In case of IEEE 802.11b for example, the maximum coding rate should be kept as low as 400 kbps while using the same parameters as our IEEE 802.11g network, e.g., local network capacity of 2,000 in volume.

### 3.3. Stimuli Adjustment

With the mechanisms we explained so far in the paper, a desired spot pattern appears and each node generates video data with the appropriate quality in accordance with the location, speed, and direction of a moving target without knowing the complete information about the whole system. However, when two or more targets are located close together, the total amount of video traffic would exceed the network capacity in that area. As a consequence, the perceived video quality considerably deteriorates for loss and delay of video data. To tackle the problem, we additionally propose the stimuli adjustment mechanism.

A basic idea is as follows. When two or more targets are close together, spots centered at them overlap with each other. If a node can detect the overlap and inform to the nodes setting the stimuli, the amount of stimuli can be adjusted so that spots become small and apart from each other. As shown in Figure 4(a), when two targets are closely located, both of concentrations of activator and inhibitor become high at the in between region. Especially, the concentration of inhibitor has a peak at the center. By using this phenomena, a node at the overlapping point detects the overlap.

At the control timing, a node compares the concentration of inhibitor of itself with those of neighboring nodes. If the inhibitor concentration is the highest at the node and it does not have a target in its observation area, it sets the NIP notification in a message it broadcasts. Now, a node receives a message. If $u/\sqrt{v}$ of the node is higher than that of a node from which it received NIP, the node has the stimulus, or any neighboring node of the node has a target, it sets NIP in a broadcast message. As a consequence, NIP follows the gradient of $u/\sqrt{v}$ and diffused stimuli toward nodes having a target.

When a node having a target receives a message with NIP, it reduces the stimulus *E* as *E* × *α* (0< *α* <1). When the targets move apart from each other and the overlap disappears, the stimulus has to be increased. Therefore, a node having a target increases the stimulus as *E* = *E* + Δ*e*, if it does not receive any NIP in the preceding control interval. The stimulus must be large enough to generate a pattern and smaller than the maximum to keep the volume. The range is determined from *v*(*E, A*). For example, with *A* = 0 4, the range is from 440 to 1,010.

## 4. Simulation Experiments

In this section, we show results of simulation experiments to verify the effectiveness of our mechanism. One hundred nodes are arranged in a 10 × 10 grid with separation of 100 meters. Parameter setting for the reaction-diffusion equation is summarized in Table 1. These parameters are chosen to satisfy the Turing condition of Equation (4). For the stimuli adjustment, *α* = 0 999 and Δ*e* = 1 are used. We determine the relationship *v*(*E*, *A*) from Figure 5, which is obtained by preliminary experiments with one stationary target. Assuming that the capacity of wireless network is 2,000 in volume, the mapping from the speed *V* to the attenuation coefficient *A* and the range of the stimulus *E* are summarized in Table 2. These values are determined from *v*(*E, A*) shown in Figure 5, so that the area of high coding rate spreads toward the direction of a moving target while keeping the total volume of a generated pattern does not exceed the capacity of a wireless network as will be verified in Section 4.2. Initially, the morphogen concentrations are set at zero.

### 4.1. Stationary Target

**Figure 6.**
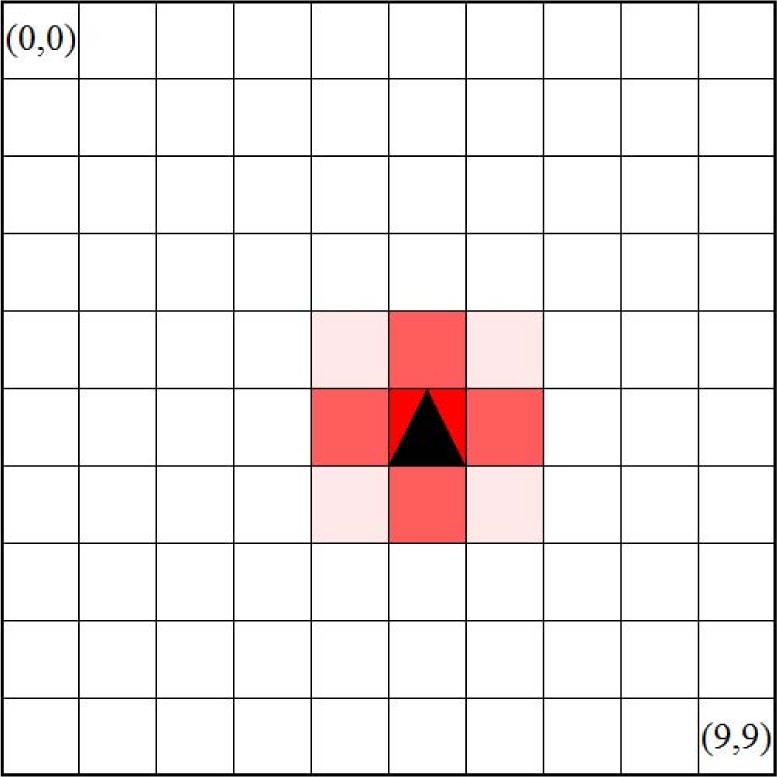
Distribution of morphogen concentrations with one stationary target.

**Figure 7.**
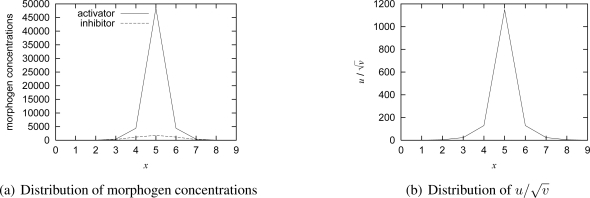
Simulation results of one stationary target.

First, we consider a scenario where there is one stationary target (*V* = 0) at the location of (5, 5) in the monitoring region (0 ≤ *x* ≤ 9, 0 ≤ *y* ≤ 9). Figure 6 illustrates the distribution of $u/\sqrt{v}$ when a pattern converges. In the figure, each square corresponds to a node or its monitoring area. The darker the square is, the higher the video coding rate and $u/\sqrt{v}$ are. A triangle indicates the location and direction of a node. As shown in the figure, a spot pattern centered at the target is self-organized. Figure 7 shows the distribution of morphogen concentrations and $u/\sqrt{v}$ on a horizontal line *y* = 5. As shown in the figure, the concentration of activator and $u/\sqrt{v}$, that is, the video coding rate is the highest at the node having the target.

**Figure 8.**
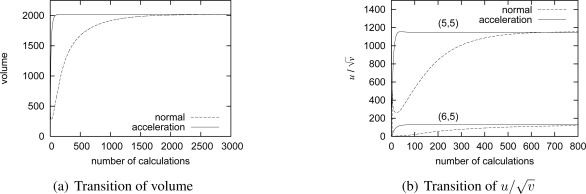
Transition of volume and video coding rate against number of calculations.

In a reaction-diffusion model, a pattern does not appear at once. Since calculation and communication require energy and time and adjustment of video coding rate must be performed in time to monitor a moving target, we have to consider the time required for pattern generation. Figure 8(a) shows the transition of the total volume against the number of calculations to show how fast a stable pattern emerges. For a pattern to converge, nodes have to calculate the reaction-diffusion equation and exchange messages about 2,800 times as a line labeled as “normal” shows. To accelerate pattern generation, we introduce an acceleration method using a larger discrete step interval Δ*t* [14]. A line labeled as “acceleration” in Figure 8(a) shows the result of acceleration by setting Δ*t* at 2.0, which is within the limit of Δ*t* = 2.32 derived from Equation (10). By the acceleration, the number of calculations is greatly reduced to 140. Now, assume that a target is moving at the speed of 4 km/h. A target passes across the observation area of 100 m × 100 m in 90 seconds. Since the control interval should be in an order of several seconds at least, the 140-times calculation is still too large to generate a pattern in time. However, it is not necessarily required for the whole pattern to converge from a practical point of view as far as a camera having a target generates the high-quality video data. Figure 8(b) shows the transition of $u/\sqrt{v}$ at a node detecting a target. For $u/\sqrt{v}$ to converge, the node needs to calculate the reaction-diffusion equation 85 times, but the concentration drastically increases to the sufficiently high value in about 30-times calculations. As nodes exchange messages and calculate the reaction-diffusion equation, further nodes eventually adjust the video coding rate as shown in Figure 8(b). A pattern is gradually generated from the center.

### 4.2. Moving Target

**Figure 9.**
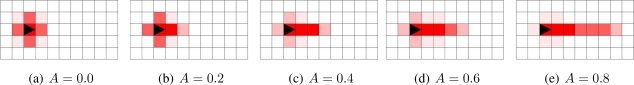
Generated patterns for a moving target.

**Figure 10.**
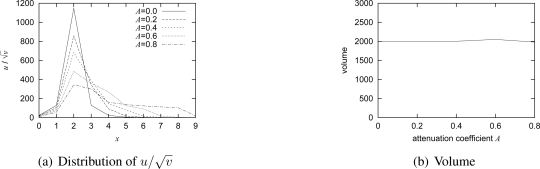
Simulation results of one moving target

Next we make a node located at (2,5) move at the speed of 1, 3, 5 and 7 km/h, which corresponds to the attenuation coefficient *A* of 0.2, 0.4, 0.6 and 0.8, respectively. Figure 9 illustrates generated patterns. Figure 10(a) shows the distribution of $u/\sqrt{v}$ for the different attenuation coefficient on *y* = 5. As can be seen, as *A*, *i.e.*, the speed of moving target, increases, the resultant pattern spreads wider and the height of peak becomes lower. As shown in Figure 10(b), the total volume is kept constant at the given capacity even when the speed changes with our stimulus setting.

### 4.3. Two Stationary Targets

**Figure 11.**
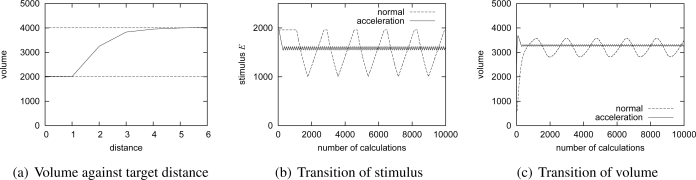
Simulation results of two stationary targets.

To verify the effectiveness of our stimuli adjustment for closely located targets, we conduct simulation experiments with two stationary targets. Figure 11(a) shows the relationship among the distance between targets and the total volume of converged pattern. Two dashed lines correspond to the volume for the case of one stationary target (lower line) and its doubled amount (upper line), respectively. The distance of zero corresponds to the case of one stationary target. As shown in the figure, the total volume is suppressed when the distance between two targets is small. In cases of the distance of one and two, intermediate nodes detect the overlap, NIP is sent to the nodes detecting targets, and the stimuli are decreased. Once the stimuli become small enough, the two nodes begin to increase the stimuli again for not receiving NIP. As a result, the stimuli fluctuate as shown in Figure 11(b), where the transition of stimulus at one of nodes having a target is depicted for the case of distance of two. It should be noted that the acceleration method also contributes to suppression of the oscillation. We see the same effect in the total volume as shown in Figure 11(c). In the case of distance of three or more, spots centered at targets do not overlap with each other and NIP is not used.

### 4.4. Multiple Moving Targets

We consider a scenario with multiple moving targets. Initially, targets which move to the random direction at the random speed from 0 to 8 km/h are located at randomly chosen nodes. At every control timing, a target changes the speed and direction with the probability of 0.005. The control interval is set at 2 seconds. At every 180/*V* control intervals, a moving target migrates to a neighboring node in the moving direction. Here, 180/*V* is the time required for a target moving at the speed of *V* km/h travels 100 meters. The discrete step Δ*t* is set at 2.0.

**Figure 12.**
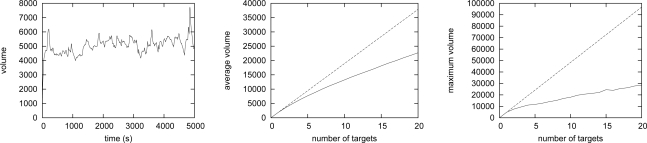
Simulation results of multiple moving targets.

Figure 12(a) depicts the transition of the total volume of the whole region with three moving targets. Depending on the distance among targets and their speed and direction, the total volume dynamically changes. One reason that the total volume exceeds 6,000, *i.e.*, the triple of the volume of a single target, is that the high concentration of activator sometimes remains behind a fast moving target. The other reason is that suppression of stimulus *E* is too slow for closely located targets moving at the speed of more than 6 km/h. We need to accelerate pattern adaptation, but it is one of future work.

Figures 12(b) and 12(c) show the relationship among the number of targets and the average and maximum volume of generated patterns, respectively. The dashed lines stand for the product of the number of nodes and the value for one target. As shown in the figures, our mechanism suppresses the traffic volume much lower by reaction-diffusion based control.

Although results are not shown, we also conducted simulation experiments on a large-size network, where 2,500 nodes are arranged in a 50×50 grid with separation of 100 meters. We verified that every node appropriately adjusts the video coding rate to monitor moving targets.

### 4.5. Influence of Parameter Setting

To see the influence of parameter setting on generated patterns, we conducted experiments on a network of 10×10 grid layout where a moving target is located at (3,5) with the speed of 5 km/h heading to the right direction.

**Figure 13.**
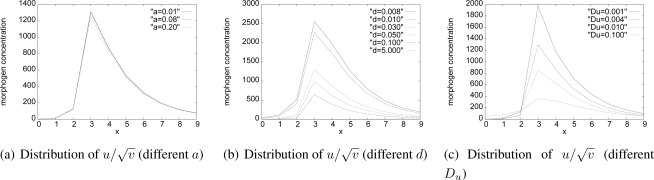
Simulation results of one moving target for different parameter values.

When we change *a* in Equation (3), the distribution of $u/\sqrt{v}$ on *y* = 5 does not change much as shown in Figure 13(a). Although not shown in figures, pattern generation is insensitive to setting of *b*, *c*, *e*, *h*, and *f*. On the contrary, *d*, *g*, *D_u_*, and *D_v_* in Equation (2) affect generated pattern very much. Figure 13(b) shows that a larger *d* decreases the height of $u/\sqrt{v}$, by decomposing the activator at the higher rate. Parameter *g* has the same effect. The larger diffusion rates *D_u_* also decreases the activator concentration by diffusing the activator mode to neighbor nodes as shown in Figure 13(c). Since $u/\sqrt{v}$ corresponds to the coding rate and further to the local channel capacity, these parameters should be carefully chosen in accordance with the targeted coding rate through preliminary numerical experiments.

These results also imply that we can control the shape and volume of pattern by dynamically changing parameters, *i.e*., *d*, *g*, *D_u_*, and *D_v_*, in accordance with the available wireless capacity and the velocity of a target. However, for this purpose all nodes have to use the same parameters and thus adaptation of parameters introduces considerable communication overhead in disseminating a new set of parameters. On the contrary, as discussed in Section 3, the amount *E* of stimulus and the attenuation coefficient *A* affects the shape and volume of pattern as well. Since adaptation of these parameters, *i.e.*, *E* and *A* to dynamically changing operational conditions can be achieved at a node independently from the others, we plan to regulate parameters *E* and *A*, while using the same and fixed set of reaction-diffusion parameters listed in Table 1 on all nodes. It further relaxes the sensitivity of pattern generation to parameter setting, although it requires preliminary experiments to obtain Table 2. We need an algorithm to dynamically adjust Table 2 to fit to the actual operational environment, but it remains one of future work.

## 5. Practical Experiments

To verify our proposal in an actual environment, we implemented the mechanism and conducted practical experiments. In this section, we give an overview of our implemented system and show results of experiments.

### 5.1. Video Coding Rate Control

**Figure 14.**
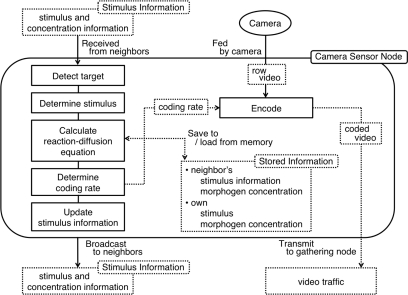
Flow of information and data in a node.

For the purpose of local logging of experimental data at each node, we used laptop PCs equipped with an IEEE 1394 camera and IEEE 802.11g wireless interface for a camera sensor node. Figure 14 shows how messages and video data are processed in a node. A node maintains information including the morphogen concentrations and stimuli on neighbor nodes and itself. At regular intervals, a node first checks whether it has a target in the observation area or not. If a target exists, a node determines the corresponding stimulus information, *i.e.*, *E* and *A*, in accordance with the velocity of the target as in Table 2. Initially, the stimulus *E* is set at its maximum limit. Next, a node calculates the reaction-diffusion Equation (8) based on the morphogen concentrations of itself and neighbors and the stimuli. The computational complexity is not high where Equation (8) is simple floating point arithmetic and can even be transformed to integer arithmetic. Then, it determines the coding rate based on the value $u/\sqrt{v}$ as in Table 3. Finally, it broadcasts a message containing its morphogen concentrations, the stimuli information, and NIP, by using UDP/IP broadcasting. The payload size is 1,649 bytes containing logging information. Since the communication and computational overhead at a node are not too high, our proposal can be implemented on low-cost visual sensor nodes with the capability of wireless communication, arithmetic computation, and video rate control.

By using the latest coding rate, a node generates MPEG-2 video data from raw video frames fed by a camera in YUV422 format. As an encoder, we used a software encoder called mpeg2vidcodec_v12 developed by MSSG (MPEG Software Simulation Group) [17] and modified it so that the coding rate could be dynamically changed during coding a MPEG-2 stream. Video data are sent to a base station by using UDP/IP unicast communication. The payload of one video data packet amounts to 1,092 bytes. Although we adopt MPEG-2 in our experiments for the purpose of proof of concept, other video coding algorithms such as MPEG-4 and other rate control mechanisms [18] such as frame dropping or temporal resolution adaptation can be applied to our proposal and we do not limit to our experimental setting. By choosing more sophisticated algorithms and mechanisms together with error concealment techniques in the deployment phase, a video surveillance system with the higher video quality and the higher tolerance to bit errors can be realized.

### 5.2. Object Detection

Although some commercially available cameras are capable of object and motion detection, in our implementation, we used a simple mechanism for the purpose of preliminary experiments and easier control. The accuracy and effectiveness of object detection are out of scope of our research.

A node detects the existence and movement of an object by comparing the difference in luminance between two successive video frames. The luminance difference is derived for each pixel (*i, j*) by subtracting the luminance *f*(*i, j*) in the current frame from the luminance *F*(*i, j*) in the preceding frame. If the number of pixels whose absolute value of luminance difference is above the threshold *x* exceeds the threshold *y*, a node considers that there is a moving object in the observation area. The moving direction of the target is estimated by comparing the average coordinates of changed pixels in the successive several frames. Since estimation of the moving speed requires the complicated image processing such as estimation of the object size and its distance, we did not implement the speed detection mechanism and used the fixed value.

### 5.3. Experimental Setting

In simulation experiments, we verified that each node could adjust their coding rate properly and the total amount of video traffic was suppressed by applying our proposal even for cases of multiple targets. However, due to time and facility limitation, the experiments in this paper were with only one target and four camera sensor nodes.

**Figure 15.**
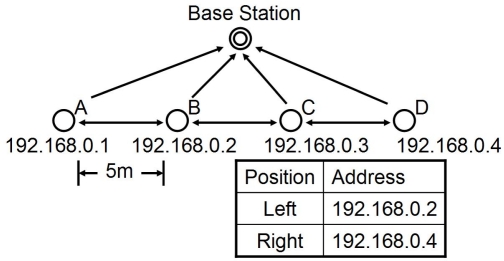
Node layout in experimental system.

We arranged four camera sensor nodes 5 m apart in a line as illustrated in Figure 15. A base station was located between nodes B and C and apart from the line. They were connected by IEEE 802.11 IBSS (Independent Basic Service Set) mode. All nodes belonged to the same IP subnet by having the same network address. Although all nodes were within the range of wireless communication, we manually configured them so that a camera sensor node could exchange messages only with a base station and its adjacent neighbors by ignoring messages from distant nodes. The control interval was set at 0.25 second. Thus, a node consumes 53 Kbps for control messages. All nodes operated asynchronously, where they broadcast messages, calculated the reaction-diffusion equation, and adjusted the coding rate at different timing. Other parameters used for the experiments are summarized in Table 1.

### 5.4. Evaluation Measures

We use packet loss rate and PSNR (Peak Signal to Noise Ratio) as measures of evaluation. The packet loss rate is defined as the ratio of the number of video data packets which are not received at a base station to the number of video data packets sent from camera sensor nodes. The PSNR is derived by the following equation.

(12) $$\mathrm{PSNR}=20\;\text{log}\;10\left(\frac{255}{\sqrt{\mathrm{MSE}}}\right)$$

MSE represents the mean square error. MSE of an image of *m* × *n* pixels is calculated the by following equation.

(13) $$\mathrm{MSE}=\frac{\sum {\left(f\left(i,j\right)-F\left(i,j\right)\right)}^{2}}{\mathrm{mn}}$$

where *F* (*i, j*) and *f*(*i, j*) are the luminance at the pixel (*i, j*) of an original image and that of a comparison image, respectively.

### 5.5. Results and Discussion

We first evaluated the performance of our implemented system and found that, to accomplish the detection, calculation, broadcasting, and coding in a real-time fashion, the spatial resolution and the frame rate should be kept as low as 320 × 240 pixels and 8 frames/sec, respectively. This results in the video rate of 1 Mbps at maximum and cannot cause congestion in the wireless network of only four nodes. Therefore, in the following, we use dummy traffic equivalent to video data of 720 × 480 pixels and 30 frames/sec in size. On the base station, loss of video data packets was monitored and recorded, from which received video data were generated by using the video coding rate determined in experiments.

**Figure 16.**
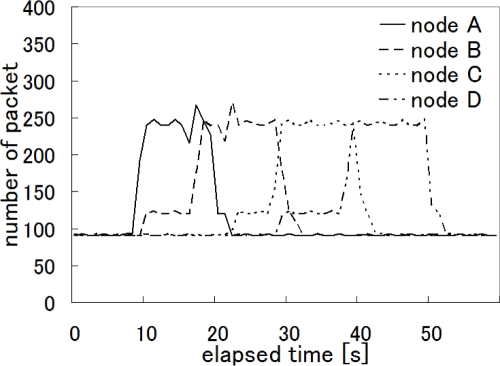
The number of emitted packets with our proposal.

Figure 16 illustrates the time variation in the number of packets emitted by the camera sensor nodes per second when our proposal was applied. As the target walked at the speed of about 1.8 km/h from the left (node A) to the right (node D), it moved from the observation area of one node to another in about 10 seconds. This corresponds to the time variation in the video traffic shown in Figure 16, where the duration that a node sent video data packets at the rate of about 250 packets/sec, *i.e.*, 2 Mbps is about 10 seconds. A node in the direction of the target movement first raised its coding rate from 0.75 Mbps to 1 Mbps, and then set it at 2 Mbps on having the target in its observation area, and finally returned to its normal rate of 0.75 Mbps when the target disappeared from the observation area.

**Figure 17.**
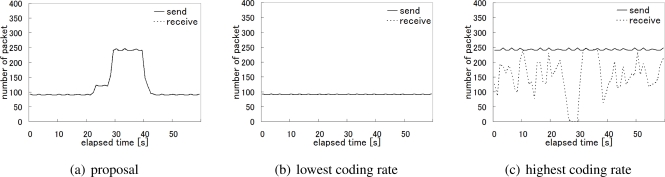
The number of sent and received packets (Node C).

Next, in Figure 17, we compare three alternatives focusing on the node C, which was located near the base station and thus affected by other traffic. As we do not see any visible gap between the number of packets sent by the node C and the number of packets received at the base station for the node C in Figure 17(a), the packet loss rate was only 0.01% for the node C during the whole experiment. Figure 17(b) shows the case of adopting the lowest coding rate, *i.e.*, 0.75 Mbps, hesitating to generate the high quality video and introduce much video traffic into the network. In this case, the packet loss rate was the lowest and 0%. Finally, the packet loss rate of the case where all nodes aggressively used the highest coding rate is shown in Figure 17(c). As shown in the figure, the considerable number of packets were lost and the loss rate was about 36.7% for overloading the wireless network.

**Figure 18.**
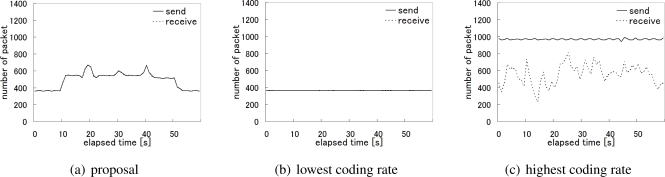
The total number of sent and received packets.

Figure 18 shows how the whole network was loaded. The figure illustrates the time variation in the total number of packets sent by all sensor nodes and the total number of packets the base station received. The number of received packets in Figure 18(c) indicates the capacity of the wireless network. It is approximately 4.65 Mbps on average. It is almost equivalent to the total video traffic when one node uses the coding rate 2 Mbps, another node does that of 1 Mbps, and the other two nodes keep the lowest at 0.75 Mbps. In our proposal, the camera sensor nodes autonomously choose the optimal coding rate allocation in accordance with the location of the target by adopting the reaction-diffusion model. The average transmission rate of video data per node is about 1.03 Mbps with our proposal. The resultant packet loss rates are 0.24%, 0.02%, and 42.5% in Figures 18(a), 18(b), and 18(c), respectively. The packet loss rates for nodes having the target in observation area are 0.03%, 0%, and 38.8%, respectively.

**Figure 19.**
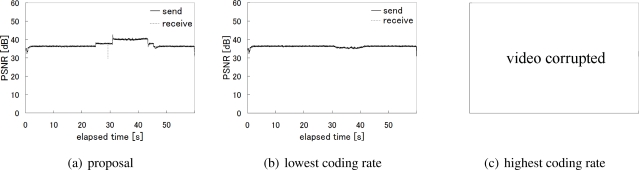
PSNR of sent and received video data (Node C).

**Figure 20.**
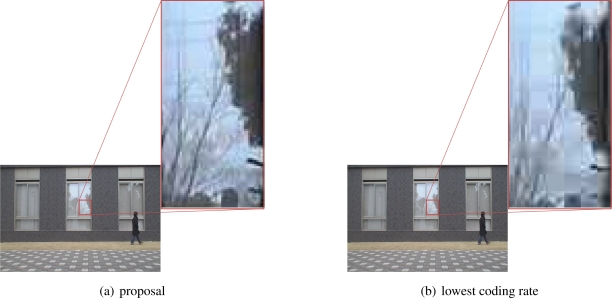
Perceived video image sent from node C at 40 second.

The high packet loss rate severely affects the perceived video quality. Figure 19 shows the time variation in the PSNR value for video data sent by the node C and that received at the base station for the node C. The PSNR is derived against original video frames captured by a camera. Since the packet loss rate was too high to decode the received video data when the highest coding rate was always used, the PSNR values are only shown for the other two cases. In the case that our proposal was adopted, the node C adjusted the video coding rate in accordance with the target movement, as we see the increase in the PSNR to 40.0 dB at about 31 second. The reason for the instantaneous decrease of PSNR at about 29 second was for loss of packets containing the header information. This can be recovered by using FEC or we can avoid loss of header information by prioritizing important packets to the others. On the contrary, although such spike-like variation does not appear in Figure 19(b), the PSNR was around only 36.7 dB. Especially when the node C had the target in its observation area, the PSNR slightly decreased to 35.5 dB for the complexity and activity of captured images. Figure 20 compares the perceived video images from the node C at 40 second for the cases of our proposal and lowest coding rate. Although the difference is not obvious, we see a finer image with our proposal.

In conclusion, with our mechanism, camera sensor nodes autonomously adjusted video coding rate to avoid congestion of the wireless network in order to satisfy application requirements on the perceived video, where a user could monitor a target at the high quality description on a display.

## 6. Conclusions

In this paper, we proposed a reaction-diffusion based autonomous control mechanism for camera sensor networks. In our mechanism, nodes periodically exchange information about the morphogen concentrations, calculate the reaction-diffusion equation, and adjust the video coding rate. By setting the stimulus at a node detecting a target and diffusing the stimulus, the video coding rate becomes high at a node with a target and nodes in the moving direction. Through simulation and practical experiments, we verified the effectiveness of our proposal. We showed that network congestion was avoided and the quality of video data from a camera having target was high.

## Figures and Tables

**Table 1.** Parameter settings for reaction-diffusion equation.

parameter	value	parameter	value
*a*	0.08	*h*	0.05
*b*	0.2	*D_u_*	0.004
*c*	0.2	*D_v_*	0.1
*d*	0.03	*M*	0.2
*e*	0.1	*N*	0.5
*f*	0.14	Δ*t*	0.1
*g*	0.06	Δ*h*	1.0

**Table 2.** Mapping from *V* to *A* and range of *E*.

*V* (km/h)	*A*	Upper limit of *E*	Lower limit of *E*
*V* = 0	0.0	1,960	830
0 *< V* ≤ 2	0.2	1,370	700
2 *< V* ≤ 4	0.4	1,010	440
4 *< V* ≤ 6	0.6	620	390
6 *< V*	0.8	360	260

**Table 3.** Mapping from concentrations of morphogens to coding rate.

$u/\sqrt{v}$	coding rate
$0<u/\sqrt{v}\geq 5,000$	0.75 Mbps
$5,000<u/\sqrt{v}\geq 10,000$	1 Mbps
$10,000<u/\sqrt{v}$	2 Mbps
